# The Origin and Cure of Syphilis

**Published:** 1894-07

**Authors:** Rufus Choate

**Affiliations:** Washington, D. C.


### THE ORIGIN AND CURE OF SYPHILIS.

Rufus Choate, M. D., Washington, D. C.

The three cases herewith presented for your consideration are confirmative of the curability of syphilis through following the law of strict Homœopathy and using high potencies. Theorizing on pet methods of treatment will not effect the work that the clear, indisputable presentation of positive cures performed will accomplish. If the words I earnestly speak to-night will bring you to a trust in that Homœopathy the master gave, if it will prevent the endeavor to suppress the local manifestations of disease, if it will guide you in the duty of exterminating the crime of syphilis, I will have received a reward far beyond the gratification of keeping secret a process, not new, alas! it is true, but which is always successful in curing that most dreadful of all diseases.

It has been the desire of those who would trace venereal diseases to their origin to ascribe their introduction to the arrival of another and a foreign people. To the Moors in Spain, for instance. But if I advance a theory showing the possibility of its origin, not infection, mind you, where it never existed before, and where there is no communication with a foreign people, you will admit, perhaps, that the time of its beginning in the history of man has no bearing in the case. Take four people, two men and two women, husband and wife, living in conjugal love, and suppose there are no other people existing. As long as the conjugality exists syphilis is an impossibility with these four people. Let convivial love, the love of hatred toward the partner in marriage, the love that prevails in hell, the love from which all diseases arise, a promiscuous love, first to the partner and then to the other, and syphilis is an inevitable result. As long as these four people lived true to each other and entertained only his or her own partner there was no danger, but when these four people led a promiscuous life, syphilis branded their evil in loathsome letters. As it was in the beginning, so it is to-day, and so it will continue to be as long as convivial love lies near the heart of man. A man or a woman married and true is pure. That man or woman going to another has already originated the disease in the body of the animus—a better name, by the way, than "astral body"—from whence all diseases come, and starts from a pure stream a foul disorder that flows through many generations of sons and daughters. Think it not necessary that the germ of the disease shall have a prior existence in the physical body. The germ is in the evil of the convivial love; purity, happiness, and the highest use are in conjugal love.

Mr. A., a gentleman of intelligence beyond the average, in the vigor of life, wealthy, with the fairest worldly prospects discovered the indiscretion of three weeks prior resulted in a chancre on the glans penis. At this time he was residing in the West, and on his return to Washington applied to me for treatment in response to a peculiar mental monition.

The chancre looked as though some one had applied a rusty, rough, notched pair of small iron pinchers to the corona of the glans penis and had viciously pulled a piece of flesh therefrom. The patient could not look down upon this dreadful place without a feeling of sickness. The beauty of the world had flown, and the Potomac River gave to this distressed and harassed mind its tempting place of oblivion. On looking at the chancre I promptly said: "There is no doubt about it; it is an indurated chancre of syphilitic origin." I know full well that certain minds of negative principle when they come to read this article will say they have learned nothing from it, or will doubt if the case was in fact syphilitic, or will add that the case should be kept under the scrutiny of their own superior wisdom for the next fifty years to prove that no secondary symptoms show themselves, rather than acknowledge that so great a disease can be cured by the use of a high potency drug and in accordance with the great, grand laws given by Hahnemann. These negatives, in their wordly-wise wisdom must burn, cut, mutilate and inject their own proprium into the case. The patient appeared to be peculiarly startled by my words, and then told me that while away from the city and he first realized the terrible condition in which he was he had a dream. In the dream he was directed to apply to me for treatment and on coming to me in the dream I had then used the very words I now repeated. When I add I had seen this gentleman only once before, and that three years ago, and I had forgotten him as completely as I have no doubt he had forgotten me we will take the dream as a peculiar mental state, which I shall not argue against, if it is going to work by sending me patients.

This consultation was October 3d, and I gave one dose Nux-vom.$^{3300}$ with instruction to protect the chancre by applying a piece of absorbent cotton. The condition of his tongue, the constipation, the anxiety, the state of his life, called for Nuxvomica. November 1st I gave Merc-sol.$^{14m}$, one dose, and Sac-lac. every three hours. November 5th, gave another dose Merc-sol.$^{14m}$ and continued Sac-lac. November 8th, his tongue became milky-white, accompanied by a feeling of sadness and despondency. I gave Anti-crud.$^{25m}$, one dose. November 11th, Merc-sol.$^{14m}$, one dose. November 14th, Merc-sol.$^{14m}$, one dose, and now the chancre was evidently changing. November 18th, when he removed the cotton there came from several minute places drops of blood. It was not due to the removal of the cotton, but was characteristic, and I gave Nitric-acid$^{1000}$. November 20th, the discharge of blood had ceased. The patient is decidedly better, hopeful, and there is an appearance as though the upper portion, or that nearest the body, is improving. I gave Merc-sol.$^{14m}$, one dose. November 23d, there is near the centre of the chancre a raised place that looks like a wart, a symptom that gives suspicion of sycosis. I prescribed one dose Thuja$^{15m}$. November 29th, improvement continues, but the wart-like appearance remains. The upper edge looks softer and less ulcerated. I gave another dose of Thuja$^{15m}$. December 1st, the patient is improving. His general condition has decidedly changed. His eye is clear, tongue is clean, and mind is healthy, bright, and cheerful. The chancre yet holds its power, and I gave Merc-sol.$^{14m}$. This acted till the 13th, and was then repeated. December 18th, the chancre is changing. The patient's general health is excellent. I gave now another, the eighth dose of Merc-sol.$^{14m}$, and on the 24th, the tenth dose, January 2d, the eleventh dose, January 21st, twelfth dose, and now the chancre has nearly healed. I have an upright man, one who has learned his lesson, and is thankful to the Physician above who has been pleased to direct his steps from death into life.

February 28th the chancre is wholly healed. There is yet the scar, but even this mark of the disease will, I assert, be wholly obliterated. I gave now the thirteenth dose of Merc-sol.$^{14m}$. I observed, March 5th, a number of minute pimples on the glans penis, and I gave Thuja$^{15m}$, one dose. March 19th the scar has two-thirds disappeared, and I want that mark wholly gone, and I gave Merc-sol.$^{14m}$, one dose. April 1st improvement continues, but scar not yet entirely gone, and until it is I shall not pronounce the case cured. I gave another dose Merc-sol.$^{14m}$. He is in better health than since his childhood, and in my patient I have found a friend whom I honor and respect. At this, the last consultation I had with him, the patient put a question of vital importance. "Doctor," he said, "does it not occasionally happen that a chancre, similar to the one I had, disappears without medical treatment?" "Yes," I answered: "I acknowledge that, and recognize the difference between the disappearance of the chancre and the cure of the patient." "Please explain," added my anxious and somewhat doubting friend. "As Moses and the magicians produced the same effect in ultimates, so the same appearance occurs in ultimates from a cure, or a suppression. There the difference ceases. In a cure the animus is well, in a suppression the animus is sick. When a chancre disappears without medical treatment, or with abortive treatment, it disappears because it is attracted within the system, and goes inward. Under pure homœopathic treatment the medicine used becomes a communicant between the disease within the body and the substance in the material universe whereby the disease flows outward and empties itself into a material external to man. Under this universal law the Creator of man makes it possible that diseases shall be removed. Beginning, as all diseases do, in the corrupt animus of man, they, through God's great mercy, show themselves in the physical body and in the cure are moved wholly outward. Only they remain in the memory of man to tell him that in case pride shall whisper words tending to glory of self that at that point you also had fallen."

My second case is a secondary. It is that of a young man of excellent position in life, owner of and ably conducting a large and well-paying establishment. Four years ago he contracted the disease. The initial injury was suppressed, and secondary symptoms appeared. He has been liberally dosed with Mercury, and has taken large quantities of Potash, and has been to the Hot Sulphur Springs. He employed the best medical ability, and his case was diagnosed syphilitic by every physician that attended him. He came to me from an allopath, who had made the attempt to obliterate the secondary ulcer that was on the abdomen near the navel. There were nodes in his head, feeling like the thickening of the skull, that were painful, and there was an ulcer on the under-side of the tongue near its tip. The ulcer on the abdomen was full of paste, prescribed by his previous physician. The ulcer was hard under the examining finger, clear-cut, deep, and dirty. I started him November 7th with one dose Merc-sol.$^{14m}$, repeated it November 15th, and the 26th. From these three doses the ulcer on the abdomen was healed, leaving a red discoloration that was not deeper than the skin, the tongue had improved, and the entire bearing of the patient had changed. Gave him Merc-sol.$^{14m}$ on December 3d, and again on the 13th. December 21st he was affected by a severe cold that called for Rumex$^{cmm}$, one dose, which made so great improvement that I did not see him again until December 27th, when he was affected by the "grip," or what I prefer calling capillaritis, and I gave him three doses Ruta$^{cmm}$. January 10th, when I next saw him, the red spot on the abdomen had become blue, the tongue was decidedly better, the nodes in the head were smaller, and the patient was bright and cheerful. I gave him the sixth dose of Merc-sol.$^{14m}$, and on the 29th the seventh dose. February 5th the ninth dose, and February 17th I was so pleased with my patient that I gave a dose of Sac-lac., and on March 14th the tenth dose of Merc-sol.$^{14m}$. The tongue is wholly well, not a trace of the ulcer remaining, and in every other respect the man is well, excepting some thickening of the skull remaining.

My third patient is a young gentleman holding a place of honor in the literary world. He had been treated by a physician proclaiming himself a homœopath, but from the method used it would be difficult to distinguish him from an able allopath. The chancre that appeared on the glans penis had been burned out in the approved "regular" manner. Massive doses of medicine had kicked the more evident and external indications of the disease deep into the system, to reappear after the system had grown accustomed to the force used with a viciousness that gave the doctor more than he could do. The patient came to me from New York. A secondary ulcer had made its *habitat* in his mouth, in the right lower jaw just back of the third molar tooth. It had been there two months, despite the doctor's endeavor to expel it. July 1st I gave him a dose of Merc-sol.$^{14m}$, and on the 4th, because of excessive dampness and coldness of feet, with other constitutional symptoms, one dose Calc-carb.$^{56m}$, and on the 18th one dose Nitric acid$^{1000}$, because the ulcer had a tendency to bleed. July 14th, the 18th, and the 25th, respectively, I gave Merc-sol.$^{14m}$, making thus far four doses of Merc. On August 7th my attention was called to a bad condition of the scalp that had existed several years prior to this infection, and I gave one dose Thuja$^{15m}$. August 14th he complained of a severe pain and great tenderness on the left side of the throat, and I gave one dose Lachesis$^{32m}$. September 14th I resumed the attack on the ulcer and gave Merc.$^{14m}$. October 3d, the ulcer has greatly changed, as has the patient. He has no doubt of a cure; feels better in every respect. I permitted the last dose to act until December 5th, when I gave another Merc. powder. By this time the ulcer has wholly disappeared. He says his health is excellent, and I find no traces of disease. March 12th, he wrote me most favorably, expressing gratitude for the benefit derived, and even thankful, considering the dangers of a life thoughtlessly spent, for the disease that woke him to the realities of existence. He came to my office from New York April 2d and I thoroughly examined him and gave him full permission to protect his life by taking a wife.

In this decision probably I will receive the adverse criticism of some, especially from him who with the first breath asserts that none of the cases given above are syphilitic, and with the next case permission to marry is far more criminal than all else, and tends to bring discredit upon Homœopathy. As if the discredit of Homœopathy is not more in the attempted suppression of the external manifestation of diseases. For my decision I have the authority of Hahnemann. When either in the body of the animus (mental symptoms) or in the physical body (the objective symptom), no indications of the disease are given, the patient is to be pronounced well, and is to be furnished with a clean bill of health, as far as that disease is concerned. In this case, I, as the physician in attendance, must pronounce judgment. It is not for another physician who has not seen the case, and is not employed, though perhaps furnished with a diagnostic acumen far superior to the one who has the case in charge, to assert the man is not cured. Preconceived notions may assert that the patient should not be thoroughly cured through such imponderable, intangible, infinitesimal quantities of medicines as that used. A medicine that Constantine Lippe carried through a large portion of his great and active life, and from whose medicine case I got it, a vial I have replenished many times, as Lippe had replenished hundreds of times, and which is not accurately Merc-sol.$^{14m}$, but is a drug that cures the subtile symptoms produced by diseases that resemble the provings of Mercury; this is all I ask. I have confirmed that vial of pellets marked Merc-sol,$^{14m}$, hundreds of times, and where Merc-sol. is indicated it never fails me. Can you do better with anything in this enigmatic world?

